# Supplementary material for: Mechanism of Action and Interaction of Garlic Extract and Established Therapeutics in Prostate Cancer
Source: Int J Mol Sci. 2025 Feb 19;26(4):1777. doi: 10.3390/ijms26041777 (PMC11855885; doi:10.3390/ijms26041777)
Supplement: Supplementary file 1 [file ijms-26-01777-s001.zip › ijms-3476028-supplementary.pdf]

International Journal of
Molecular Sciences

MDPI

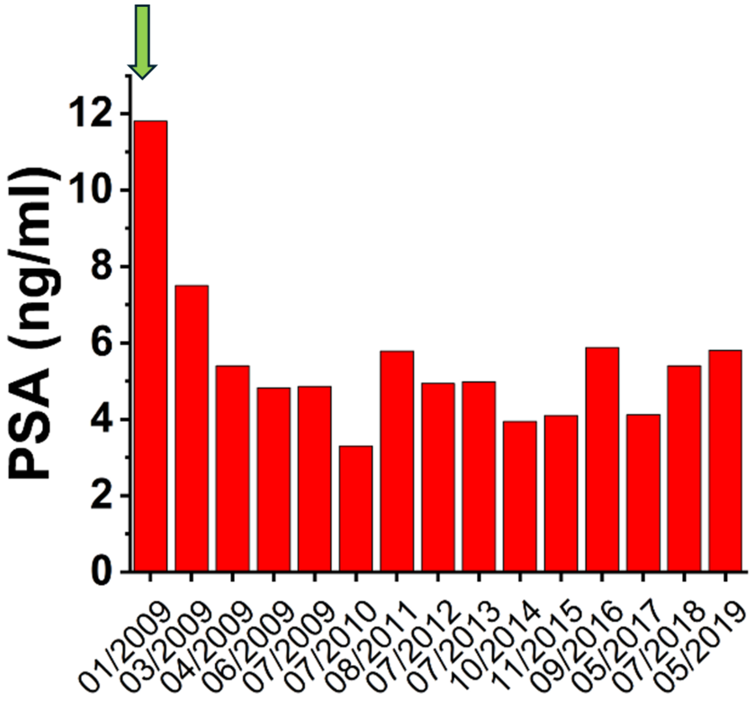

Supplementary Figure S1. PSA course of case report after GE intake. Illustrated is the course of the PSA in ng/mL after blood analysis of the case study over 10 years after the start of GE intake (green arrow).

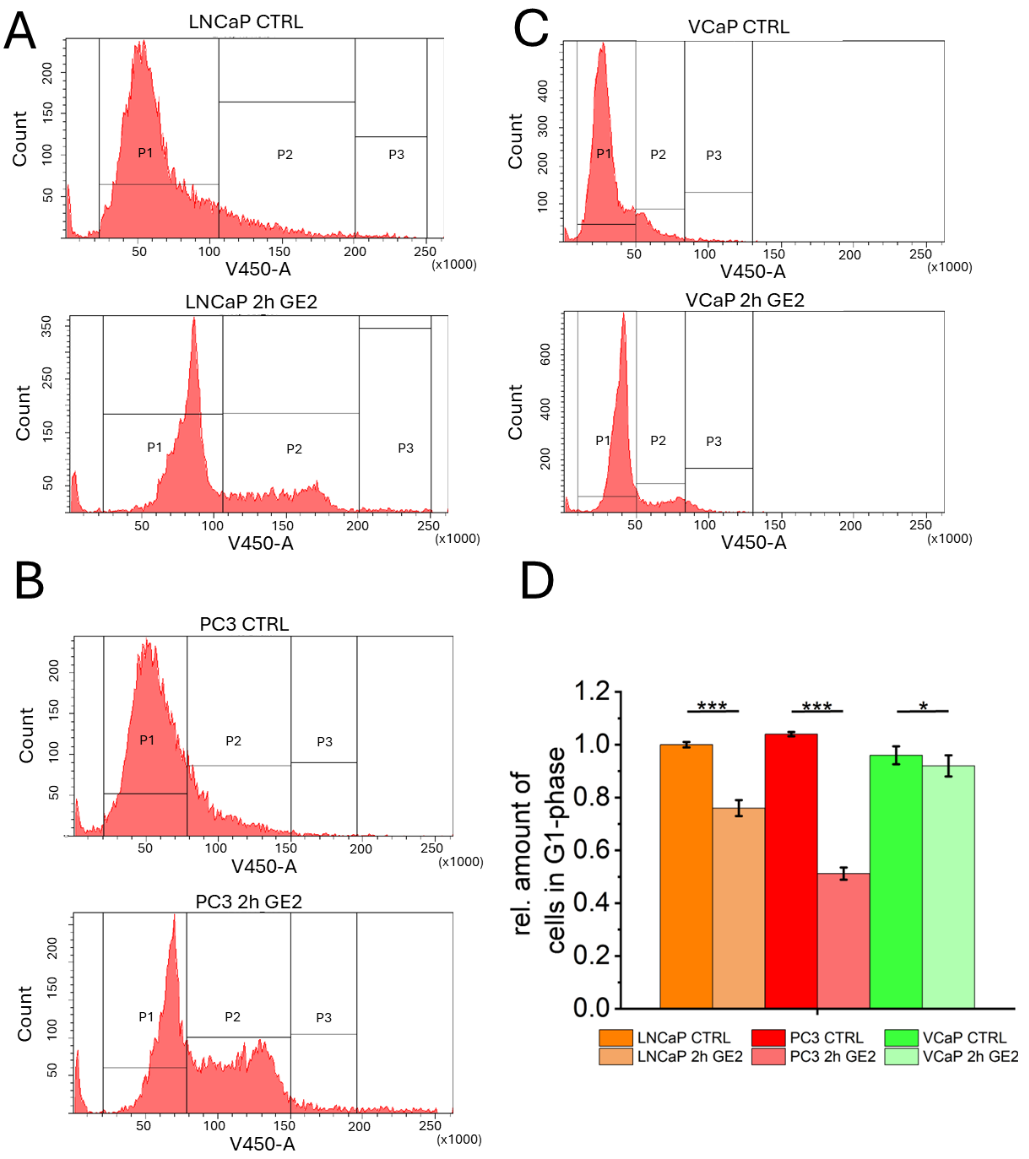

Supplementary Figure S2. **Cell cycle analysis of GE-treated PCa cells**. The cell cycle of different PCa cell lines (LNCaP, PC3, and VCaP) was quantified using nuclei staining. Histogram(A–C) display the cell cycle profiles for all PCa cells, with P1 representing the G1 phase, P2 the S-phase, and P3 the G2/M phase in control samples (CTRL) as well as in samples treated with GE2 for 2 h. In (D), the GE-induced changes in the proportion of cells in the G1 phase following GE2 treatment for 2 h are shown in a comparative analysis. The statistical significances are indicated by asterisks (*: $p < 0.05$; **: $p < 0.01$; ***: $p < 0.001$).

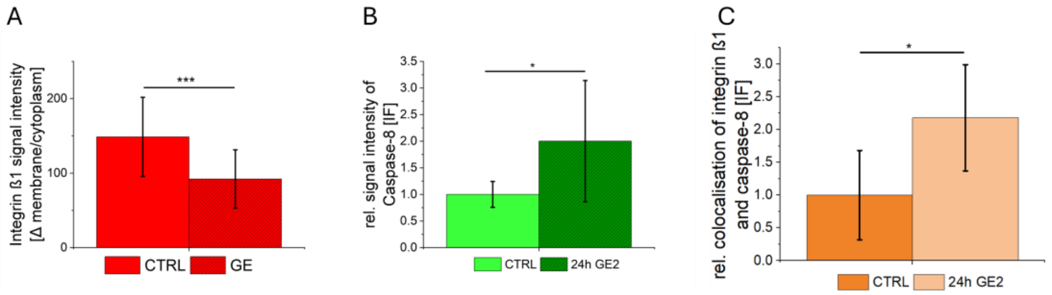

Supplementary Figure S3. Quantification of Integrin β1 intensity between membrane and cytoplasm (Δ membrane/cytoplasm) in control samples (CRTL) and GE treated samples (A). The analysis was performed using cell profile analysis of immunefluorescence against Integrin β1. Quantification of Caspase-8 signal intensity (B) and colocalization of Integrin β1 and Caspase-8 indicate alternative apoptosis induction by Integrin β1 and Caspase-8 interaction (C). The statistical significances are indicated by asterisks (*: $p < 0.05$; **: $p < 0.01$; ***: $p < 0.001$).

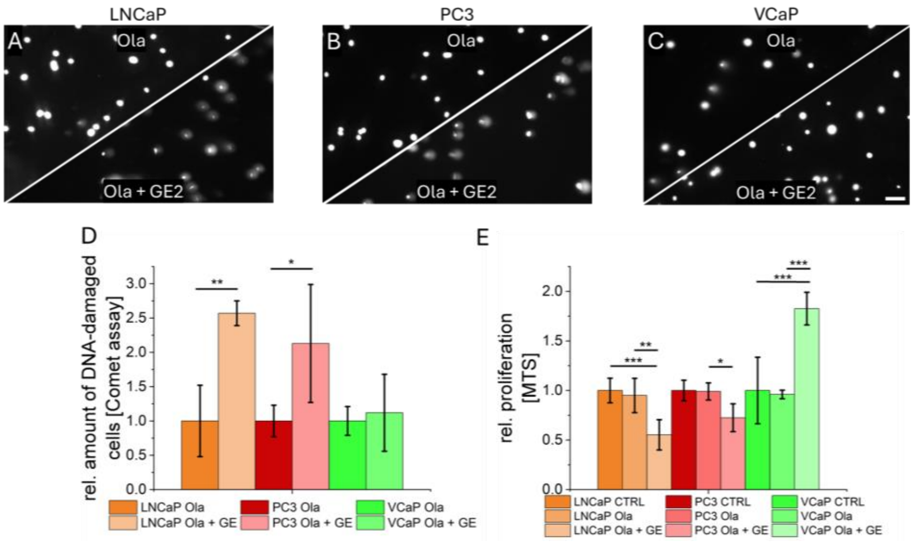

Supplementary Figure S4. The DNA damage was characterized using the Comet assay after administration of PARPi (Olaparib/Ola) alone and simultaneous GE administration (+ GE2) in LNCaP (A), PC3 (B), and VCaP (C) cells. The resulting number of DNA-damaged cells was quantified (D), and an MTS assay was conducted to investigate cell proliferation following single therapy and supplemented GE administration (E). The statistical significances are indicated by asterisks (*: $p < 0.05$; **: $p < 0.01$; ***: $p < 0.001$). Scalebar = 100 μm.

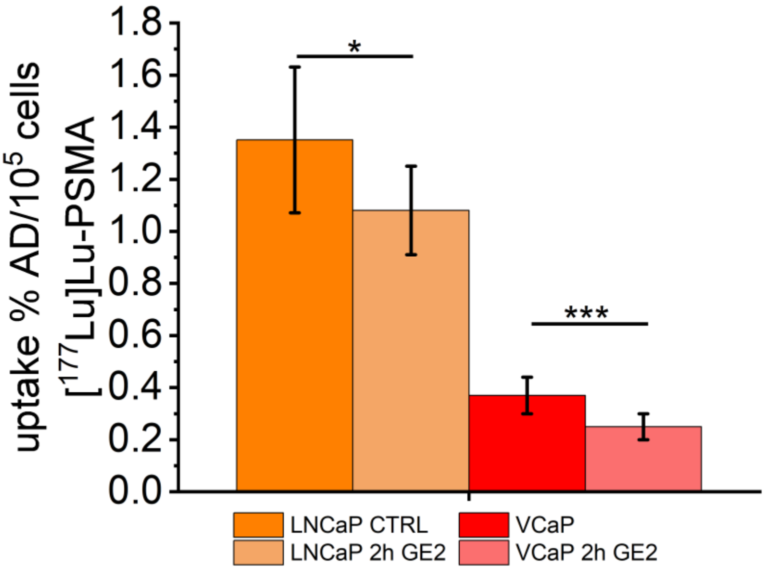

Supplementary Figure S5. Quantification of ^{177}Lu-PSMA-617 uptake. The uptake of ^{177}Lu-PSMA-617 was compared between control samples (CTRL) and GE2 treated samples for 2 h. Uptake was normalized to the respective cell numbers of each sample and expressed as % applied dose (AD). The statistical significances are indicated by asterisks (*: $p < 0.05$; **: $p < 0.01$; ***: $p < 0.001$).
